# Hypophosphatemic rickets: An unexplained early feature of craniometaphyseal dysplasia

**DOI:** 10.1016/j.bonr.2023.101707

**Published:** 2023-08-17

**Authors:** Julio Soto Barros, Demetrios Braddock, Thomas O. Carpenter

**Affiliations:** a Department of Pediatrics, Faculty of Medicine, University of Concepcion, Chacabuco esquina Janequeo S/N, 4070106 Concepcion, Chile; b Las Higueras Hospital, Alto Horno 777, 4270918 Talcahuano, Chile; c Department of Pediatrics (Endocrinology), Yale University School of Medicine, PO Box 208064, New Haven, CT 06520-8064, USA; d Department of Pathology, Yale University School of Medicine, New Haven, CT 06510, USA

**Keywords:** Craniometaphyseal dysplasia, Rickets, Hypophosphatemia

## Abstract

Craniometaphyseal dysplasia (CMD) is an infrequently occurring skeletal dysplasia often caused by a mutation in *ANKH*. The most common features are early and progressive hyperostosis of craniofacial bones, which may cause obstruction of cranial nerves, and metaphyseal flaring of long bones. Rarely, rickets has been associated with CMD, occurring early in the course of the disease. We report an infant with CMD who presented with elevated serum alkaline phosphatase activity and low serum phosphorus at age 1 month and radiographic changes of rickets at 3 months of age. Further biochemical investigations revealed a high tubular reabsorption of phosphate and suppressed FGF23 level congruent with a deficit of phosphorus availability. Therapy with phosphorus was started at 4 months of age; calcitriol was subsequently added upon emergence of secondary hyperparathyroidism. A heterozygous pathogenic variant in *ANKH* c.1124_1126del (p.Ser375del) was identified. At 19 months of age therapy was discontinued in view of the corrected biochemical profile and radiographic improvement of rickets. ©The Authors. All rights reserved.

## 1 Introduction

Craniometaphyseal dysplasia is a rare disorder characterized by progressive hyperostosis of the craniofacial bones and metaphyseal flaring of long bones. Thickening of the skull may lead to foraminal stenosis causing facial palsy, hearing loss, blindness and increased intracranial pressure due to narrowing of the foramen magnum (Dutra et al., 2012; Wu et al., 2016).

CMD can be inherited in autosomal dominant or recessive fashion. The autosomal dominant form is observed more frequently and is due to mutations in *ANKH*; de novo mutations have also been sporadically described (Dutra et al., 2012; Wu et al., 2016).

*ANKH* encodes a protein located primarily in the plasma membrane, referred to as ANK, which has been considered to have a role in the transport of inorganic pyrophosphate (PPi), and more recently has been shown to be involved in the cellular efflux of adenosine triphosphate (ATP). Extracellular ATP can be converted into AMP and PPi by the ectoenzyme ectonucleotide pyrophosphatase/phosphodiesterase 1 (ENPP1) (Szeri et al., 2022). PPi is a potent inhibitor of mineralization and may play a role in the pathogenesis of the disorder. Known mutations in *ANKH* causing CMD include missense substitutions causing single amino acid substitutions in the ANK protein, insertions, or in frame deletions within the C-terminal region (Kanaujiya et al., 2018). The mechanisms by which these mutations trigger the phenotype of CMD are not fully understood. Initially, dysfunctional osteoclastogenesis with impaired expression of the vacuolar proton pump in the osteoclast was described (Chen et al., 2009; Yamamoto et al., 1993). Subsequently, a role for ANK in osteoblastic differentiation was reported (Chen et al., 2011; Kirsch et al., 2009;). In vitro experiments with the more frequently observed ANK mutant proteins (Phe377 del, Ser375del) have reaffirmed these findings (Chen et al., 2017) and have further shown the rapid degradation and aberrant localization of ANK in the cytoplasm (Kanaujiya et al., 2018), delaying or abolishing its trafficking to the plasma membrane (Vijen et al., 2020). In addition to its function as a plasma membrane ATP transporter, a role for ANK in clathrin-mediated Golgi/endosomal trafficking has been described (Seifert et al., 2016). Thus, aberrant localization in the cytoplasm might contribute to the pathogenesis of CMD.

Elevation of serum alkaline phosphatase activity has been noted in some cases of CMD (Cheung et al., 1997; Chida et al., 2011; Dutra et al., 2012; Fanconi et al., 1988; Key Jr et al., 1988; Richards et al., 1996; Sheppard et al., 2003; Wu et al., 2016; Wu et al., 2021; Yamamoto et al., 1993). Less frequently, elevations in circulating PTH, hypocalcemia (Fanconi et al., 1988; Sheppard et al., 2003), and hypophosphatemia (Dutra et al., 2012; Fanconi et al., 1988; Wu et al., 2016) have been observed, with concomitant radiographic findings of rickets (Fanconi et al., 1988; Wu et al., 2016). We present here a patient with CMD who manifested rickets very early in her course, indicating early clinical features of CMD, and we compare her management and course to that of previously published cases with abnormalities in mineral metabolism. Written inform consent was provided by the parents of the child.

## 2 Case

**Fig. 1:**
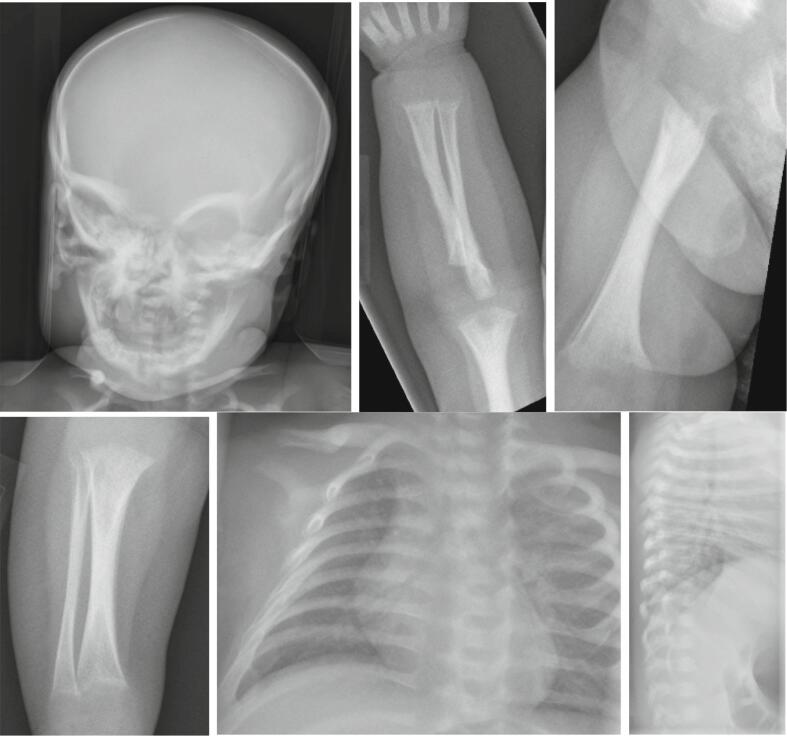
Skeletal survey at 3 months of age. Findings of mild sclerosis of facial bones, metaphyseal widening and flaring of the distal parts of the long bones and cupping at the end of radius, ulna, tibia, fibula and ribs were noted.

A 3 1/2 month-old girl was referred to our bone center for management of craniometaphyseal dysplasia. She was born after an uncomplicated pregnancy of 38 weeks with a birth weight of 2.8 kg (33rd percentile, Z-score −0.43) and length of 48 cm (67th percentile, Z-score 0.43). She was initially solely breastfed. A left facial palsy was initially noted which progressed to bilateral involvement, with predominance on the right-side. No intracranial lesions were seen on magnetic resonance images. Jaundice persisted through week 7 of life. Earlier investigations had revealed, that at 1 month of age, elevation of serum alkaline phosphatase activity was evident in concert with low serum phosphorus (3.0 mg/dL; normal range 3.8–6.5), and low to normal serum calcium (8.8 mg/dL; normal range 9.0–11). A subsequent skeletal survey at 3 months of age revealed mild sclerosis of the facial bones and skull base. Furthermore, multiple rachitic changes were evident, including metaphyseal widening and flaring of the distal femur, as well as cupping of the distal tibia, fibula, radius, ulna and ribs (Fig. 1). Computed tomography confirmed hyperostosis of the mandible, maxilla and skull base, with narrowing of the internal auditory canals and under-pneumatization of the mastoid, maxillary and ethmoid sinuses.

At our first evaluation (age 3.5 months), her height was 57.5 cm (4th percentile, Z-score −1.73) and weight was 5.5 kg (17th percentile, Z-score −0.95). She was continuing to breastfeed, and received supplemental vitamin D (400 IU daily). She had a prominent forehead, a small anterior fontanelle, hypertelorism, and asymmetric signs of bilateral facial palsy, with greater involvement on her right than left side. Laboratory investigation revealed an extremely low serum phosphorus (1.6 mg/dL, normal range 3.0–7.0), normal serum calcium (9.8 mg/dL, normal range 8.8–10.2 mg/dL), marked elevation of serum total alkaline phosphatase activity (3865 U/L, normal range 80–380) and bone specific ALP (1230 μg/L, normal range 24–124 μg/L), normal PTH (28 pg/mL, normal range 15–65) and normal 25 hydroxy-vitamin D (24 ng/mL, normal range 20–50). These findings were suggestive of a phosphopenic form of rickets. Further studies to assess renal handling of phosphate revealed a tubular reabsorption of phosphate (TRP) >96 %, and the FGF23 was below the limit of detection (<14 pg/mL), excluding FGF23-dependent hypophosphatemia. Urinary pyrophosphate was undetectable.

In view of biochemical abnormalities consistent with a primary defect of phosphorus availability and radiographic findings of rickets, elemental phosphorus, 15 mg four times a day (11.2 mg/kg/day) was initiated. After one month of this regimen, the serum phosphorus increased to the low normal range (4.7 mg/dL, normal range 4.0–8.0), with a modest decrease of alkaline phosphatase activity. An increased serum PTH level was now evident, prompting addition of calcitriol (0.06 μg twice daily; 21 ng/kg/day) to the regimen. Further increases in dose were required to counter the continued elevation in PTH (Table 1). Eventually, a heterozygous pathogenic variant in *ANKH* c.1124_1126del (p.Ser375del) was identified.

**Table 1:** Biochemical evaluations at diagnosis and follow-up.

Age	1.7 months	4.4 months	5.2 months	5.6 months	7.9 months	8.5 months	10 months	14 months	19 months	20 months	25 months
Total daily dose of phosphorus (mg)		60	105	105	150	150	90	45	Suspended		
Total daily dose of calcitriol (mcg)				0.12	0.16	0.2	0.25	0.16	Suspended		
Calcium (mg/dL)	8.8 (9.0–11)	9.8 (8.8–10.2)	9.7 (8.7–10.5)	9.4 (8.7–10.5)	9.7 (8.8–10.2)		9.9 (8.7–10.5)	10.3 (8.8–10.2)	10.0 (8.8–10.2)	9.9 (8.5–10.6)	10.1 (8.8–10.2)
Phosphorus (mg/dL)	3.0 (3.8–6.5)	1.6 (3.0–7.0)	4.7 (4.0–8.0)	3.3 (4.0–8.0)	2.6 (3.0–7.0)	4.2 (4.0–8.0)	4.8 (4.0–8.0)	5.2 (3.5–6.5)	4.6 (3.5–6.5)	5.4 (4.0–8.0)	5.0 (3.5–6.5)
Alkaline phosphatase (U/L)	2194 (38–400)	3865 (80–380)		3068 (104–450)	2287 (80–380)	1584 (100–334)	978 (100–334)	491 (100–350)	482 (100–350)	491 (117–311)	423 (100–350)
Alkaline phosphatase, bone specific (mcg/L)		1229.7 (25.4–124)									
PTH (pg/mL)		28.5		140 (7–58)	67.7 (15–65)	130 (7–58)	241 (7–58)	78.8 (15–65)	33.6 (15–65)		79.1 (15–65)
Vitamin D, 1,25 (OH)_2_, total (pg/mL)		258			365						
25 hydroxy vitamin D (ng/mL)		24 (20–50)			25 (20–50)			39 (20–50)	42 (20–50)		34 (20–50)
FGF23		<14 pg/mL (≤52)									
Collagen type I C-telopeptide (CTx), (pg/mL)		1040 (600–1700)			958 (600–1700)				1081 (600–1700)		1324
Procollagen I N terminal propeptide (P1NP) (mcg/L)		>2500 (600–3000)			1940 (600–3000)				1170 (600–2000)		1080
TRP %		≥96 % (85–95)			≥99 % (85–95)			82 % (85–95)			
TMP/GFR (mg/dL)		≤1.53 (3.16–6.19)			≤2.57 (3.5–5.82)			4.25 (3.25–5.51)			
Urinary calcium/creatinine ratio		2.6 (<0.86)			<0.1			0.4 (<0.6)			

Normal reference ranges (age-specific) are shown in parentheses after the values (Brodehl, 1994; Chubb et al., 2023; Stark et al., 1986).

**Fig. 2:**
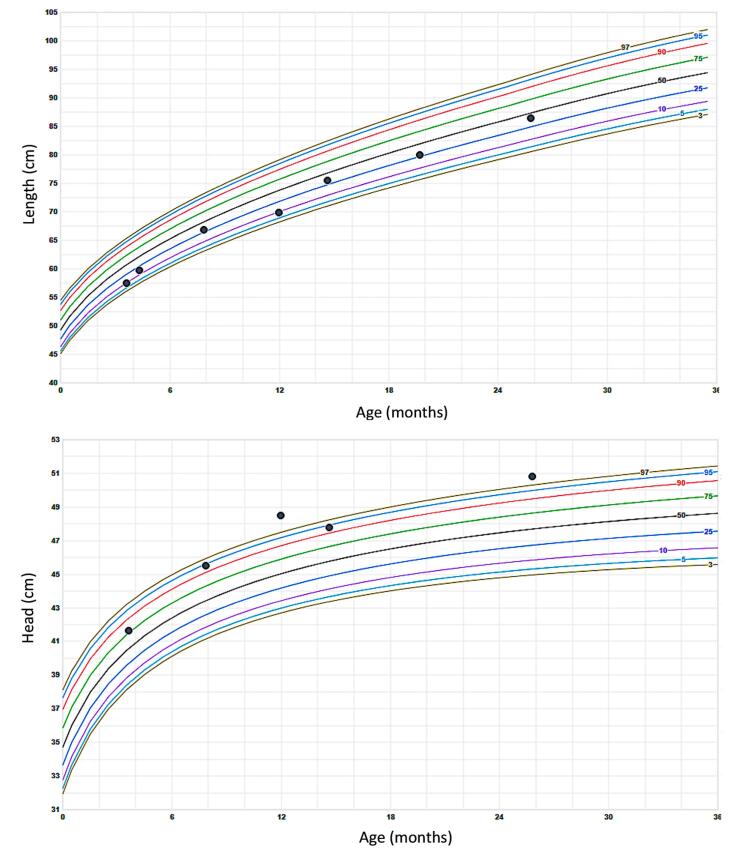
Height and head circumference growth charts.

**Fig. 3:**
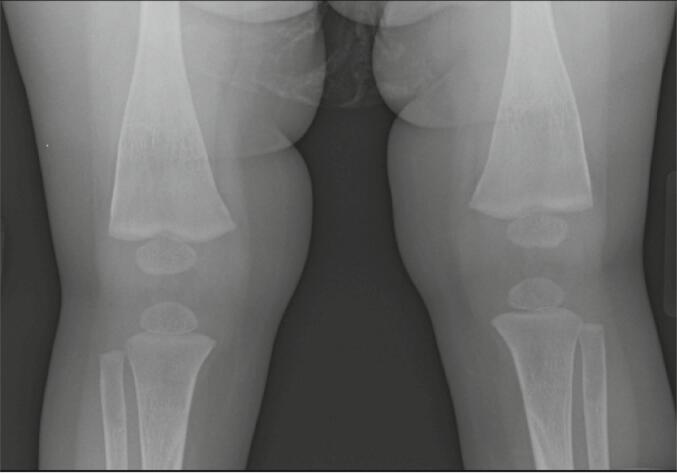
Radiographic study of the knees at 22 months of age, showing resolution of rachitic findings in distal femurs.

**Fig. 4:**
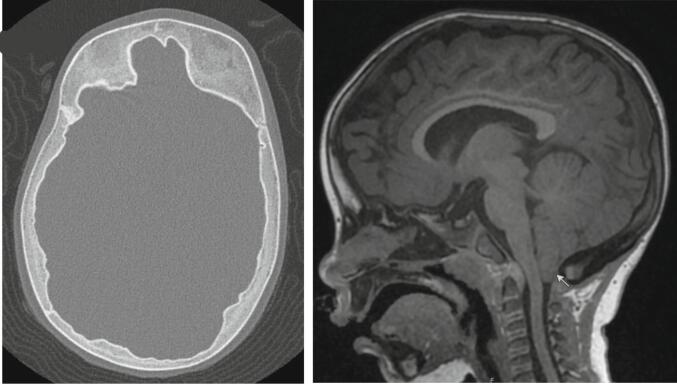
Computed tomography (CT) and magnetic resonance images (MRI) performed at age 15 months old. CT revealed fusion of the coronal sutures and MRI showed the presence of Chiari malformation type 1 (marked by a white arrow).

At age 8 months she underwent facial nerve decompression. At 15 months of age her doses of both calcitriol and phosphorus were gradually reduced, and with progressive monitoring, they were discontinued at age 19 months in view of excellent linear growth (Fig. 2), normalization of the biochemical alterations, and radiographic improvement of the metaphyseal lesions (Fig. 3). Nevertheless, an increase in head circumference has been observed during her follow-up (Fig. 2) and at age 25 months mild craniosynostosis and Chiari malformation type 1 was detected by CT and MRI, respectively (Fig. 4). She was evaluated by neurosurgical services, and no intervention has been required to date.

## 3 Discussion

This report details a female infant with CMD and hypophosphatemic rickets who was successfully managed with oral phosphorus and calcitriol. Several patients with CMD and elevation in serum alkaline phosphatase and/or PTH are described (Table 2), but hypocalcemia (Fanconi et al., 1988; Sheppard et al., 2003), hypophosphatemia (Dutra et al., 2012; Fanconi et al., 1988; Wu et al., 2016) and rickets (Fanconi et al., 1988; Wu et al., 2016) have only rarely been reported.

**Table 2:** Cases with hyperphosphatasemia and hyperparathyroidism.

Author/year	Age at diagnosis (months)	Sex	Cranial nerve affected	ALP	PTH	Ca	Pi	Rickets	Inheritance or FH	Genetic study	Medical treatment	Biochemical response to therapy	Neurological/skeletal response to therapy	Surgical treatment
Fanconi, 1988	11	F	II, VII, VIII	↑↑↑	↑	↓	↓	+	No FH	No	Calcitonin	Yes	No	Bifrontal craniectomy, decompression of optical nerves
Yamamoto, 1993	12	M	VII	↑↑↑	N	N	N	−	No FH	No	No			No
Richards, 1997	18	F	II, VII, VIII	↑?				−		No	Low calcium diet and calcitriol	No	No	Choanal stenosis, dracryocystorhinostomy, grommets insertion
	3	M	II, VII, VIII	↑?				−		No	Low calcium diet and calcitriol, somatostatin	No	No	Grommets insertion, to improve facial appearance
	24	F	VII, VIII	↑				−	FH +	No	Low calcium diet and calcitriol	Yes	No	Adenotonsillectomy, grommets insertion
Cheung, 1997	At birth	M	VII	↑↑↑	↑	N	N	−	No FH	No	No			Choanal stenosis
Key, 1988	2.5	M	VII	↑	↓	N	N	−	No FH	No	Low calcium diet and calcitriol		Yes, partial	No
Sheppard, 2003	5	F	VII, VIII	↑↑↑	↑	↓		−	No FH	No	Calcium and vitamin D	Yes (PTH)	No	Grommets insertion
Chida, 2011	7	F	VII	↑↑↑		N	N	−		No	No			Choanal stenosis
Dutra, 2012	8	F	II, VII, VIII	↑↑↑		↑	↓	−	De novo	*ANKH* c853-870del (pV285-Y290del)	No			No
Bo Wu, 2016	7	M	VII	↑↑↑	↑	N	↓	+	AD	*ANKH* c.1124_1126delCCT (p.S375del)	Calcium, vitamin D and calcitriol	Yes (PTH)	Yes, partial	Choanal stenosis
Jia-Li Wu, 2021	17	M	VIII	↑	N	N	N	−	De novo	*ANKH* c.1129_1131del (p.Phe377del)	Low calcium diet	Yes	Yes, partial	No

↑,↑↑,↑↑↑: 1, 2 or 3 or more times increase the upper limit of the normal range for alkaline phosphatase activity, respectively; ↑?: value of ALP was not reported; + or −: presence or absence of rickets; FH: family history; white space: no information available.

No clear explanation for the hypophosphatemia, in the context of this patient's CMD is evident, although that feature alone could result in the hyperphosphatasemia and rickets. *Ank* knock-out mice show the cranial phenotype of CMD, but do not manifest flared femurs nor massive jawbones. In contrast these changes are observed in *Ank* knock-in mice in which the Phe377del mutation has been introduced. Thus the pathogenesis of CMD appears to be far more complex than that predicted by *Ank* loss-of-function alone, and potentially attributable to the effects of specific alterations in the ANK protein (Kanaujiya et al., 2018). Unlike wild type cells in which the ANK protein is localized to plasma membrane, endoplasmic reticulum, Golgi apparatus and lysosomes, in cells harboring F377del or S375del mutations, ANK is found primarily in the cytoplasm (Kanaujiya et al., 2018; Vijen et al., 2020). In vitro studies in a chondrocyte line transfected with the S375del variant, identified in our patient, show decreased ePPi and increased mineralization (Vijen et al., 2020). It is possible that hypocalcemia and resultant secondary hyperparathyroidism result from this defect (Wu et al., 2016). Another consideration suggested by the very elevated increase in circulating 1,25(OH)_2_D might be vitamin D resistance, although we believe the normal calcium and PTH levels are not consistent with this diagnosis. Rather, the elevated 1,25(OH)_2_D level may be explained by the very low circulating FGF23 level. In any case, therapy with calcitriol and/or mineral supplementation has been associated with correction of the elevated circulating PTH level in one case (Wu et al., 2016). The elevation in circulating P1NP may reflect an accelerated anabolic phase of bone turnover as well (Chubb et al., 2023). Other therapeutic strategies for CMD have employed a low calcium diet to promote a hypocalcemic state along with calcitriol to generate osteoclast recruitment/activation. This approach has been associated with partial improvement of facial nerve paralysis, relief of nasal obstruction and decreased density of the skull (Key Jr et al., 1988; Richards et al., 1996; Wu et al., 2021). Administration of calcitonin was associated with resolution of hyperphosphatasemia and rickets in one case (Fanconi et al., 1988). Transient hypophosphatemia has been reported in mice (Chen et al., 2011) and in 3 patients with CMD (Fanconi et al., 1988; Dutra et al., 2012; Wu et al., 2016). FGF23 does not appear to mediate the hypophosphatemia, either in mice (Liu et al., 2016) or in the one patient where studied (Wu et al., 2016), and in whom phosphaturia was attributed to elevated PTH levels. Nonetheless, supplementation with high phosphorus diet has not improved the skeletal phenotype in mice (Liu et al., 2016). Conversely, a low-phosphorus diet, attenuated the craniofacial hyperostosis in ANK F377del knock-in mice (Fujii et al., 2020).

As mentioned above, two CMD patients have been reported with associated rickets (Fanconi et al., 1988; Wu et al., 2016;); as with our case, both children manifest hypophosphatemia, and also demonstrated hypocalcemia (Fanconi et al., 1988; Wu et al., 2016). Such a picture may be analogous to that in infantile osteopetrosis where low blood mineral concentrations occur in the setting of imbalanced bone formation and resorption and may result in so called “osteo-petro rickets” (Wu et al., 2017). Differences in the role of ANK across various skeletal sites and accompanying discordant ossification patterns may potentially explain the distinct skeletal phenotype of CMD (Fujii et al., 2020). Whether the course of the typical metaphyseal lesions seen in CMD reflects the early development of rickets is not known. If so, then early monitoring for rachitic changes and mineral disturbances may be useful as to identify the need for early treatment. However, it seems prudent to carefully monitor such therapy as to avoid promotion of excess mineralization of the craniofacial bones.

Surgical intervention may be indicated for decompression of obstructed cranial nerve foramina to slow progression of facial paralysis, as was performed in our case. Such measures may also be offered to limit progression of visual and auditory disturbances, or to improve facial appearance (Chen et al., 2011). Craniosynostosis (Fanconi et al., 1988), Chiari malformation type 1 and syringomyelia (Cai et al., 2008; Tanaka et al., 2013) may also occur in CMD; our case was found to manifest these features, however surgical intervention has not been deemed necessary to address these issues at this time. Given the spectrum of clinical complications of CMD, a multidisciplinary team including ophthalmology, otolaryngology, neurosurgery, and craniofacial surgery services offers the optimal management of these cases (Cai et al., 2008).

In summary, CMD is infrequently associated with rickets, usually early in the course. Although it resolves with typical therapies, a clear pathophysiologic explanation for this finding is not apparent. Clinicians should be aware of this feature of the disease, for diagnostic purposes and to limit the degree of skeletal complications in these patients.

## CRediT authorship contribution statement

**Julio Soto Barros:** Writing – original draft, Investigation, Formal analysis. **Demetrios Braddock:** Writing – review & editing, Investigation, Formal analysis. **Thomas O. Carpenter:** Writing – review & editing, Supervision, Investigation, Conceptualization.

## Declaration of competing interest

The authors declare the following financial interests/personal relationships which may be considered as potential competing interests: D. Braddock reports a relationship with Inozyme Pharma that includes: consulting or advisory, equity or stocks, and funding grants. T. Carpenter reports a relationship with Inozyme Pharma that includes: consulting or advisory.

## Data Availability

The authors do not have permission to share data.
